# Calculated Third Order Rate Constants for Interpreting the Mechanisms of Hydrolyses of Chloroformates, Carboxylic Acid Halides, Sulfonyl Chlorides and Phosphorochloridates

**DOI:** 10.3390/ijms160510601

**Published:** 2015-05-08

**Authors:** T. William Bentley

**Affiliations:** Chemistry Unit, Grove Building, School of Medicine, Swansea University, Swansea SA2 8PP, Wales, UK; E-Mail: t.w.bentley@swansea.ac.uk; Tel.: +44-1792-295-262; Fax: +44-1792-295-554

**Keywords:** solvolysis, substituent effects, solvent effects, acid chlorides, reaction mechanisms

## Abstract

Hydrolyses of acid derivatives (e.g., carboxylic acid chlorides and fluorides, fluoro- and chloroformates, sulfonyl chlorides, phosphorochloridates, anhydrides) exhibit pseudo-first order kinetics. Reaction mechanisms vary from those involving a cationic intermediate (S_N_1) to concerted S_N_2 processes, and further to third order reactions, in which one solvent molecule acts as the attacking nucleophile and a second molecule acts as a general base catalyst. A unified framework is discussed, in which there are two reaction channels—an S_N_1-S_N_2 spectrum and an S_N_2-S_N_3 spectrum. Third order rate constants (*k*_3_) are calculated for solvolytic reactions in a wide range of compositions of acetone-water mixtures, and are shown to be either approximately constant or correlated with the Grunwald-Winstein *Y* parameter. These data and kinetic solvent isotope effects, provide the experimental evidence for the S_N_2-S_N_3 spectrum (e.g., for chloro- and fluoroformates, chloroacetyl chloride, *p*-nitrobenzoyl *p*-toluenesulfonate, sulfonyl chlorides). Deviations from linearity lead to U- or V-shaped plots, which assist in the identification of the point at which the reaction channel changes from S_N_2-S_N_3 to S_N_1-S_N_2 (e.g., for benzoyl chloride).

## 1. Introduction

Solvolyses involve reactions of a solvent nucleophile (e.g., water or alcohol) with a suitable substrate (e.g., of general formula RX, where R is an alkyl residue and X is an electronegative group) [1]. To aid the solubility of RX in water, an organic cosolvent (e.g., acetone) is often added. An understanding of the factors influencing the reactivity of RX can be gained by studying the dependence of logarithms of observed pseudo-first order rate constant (log *k*_obs_) on empirical parameters for substituent and/or solvent effects [2]. These are examples of linear free energy relationships (LFER), from which reaction mechanisms can be proposed and effects of changing reaction conditions (e.g., cosolvents) could be predicted [3,4].

**Figure 1:**
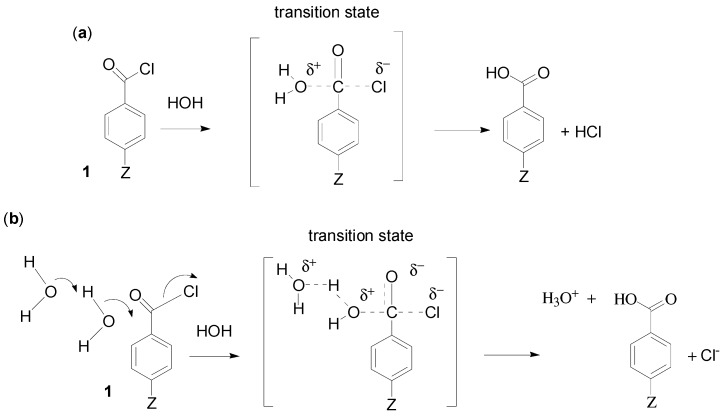
Possible reaction mechanisms for solvolyses of *p*-Z-substituted benzoyl chlorides (**1**): (**a**) The S_N_1-S_N_2 (cationic) reaction channel in which one or two molecules undergo covalency change to varying extents; (**b**) The S_N_2-S_N_3 third order (addition-elimination) reaction channel in which three molecules undergo covalency change to varying extents.

A useful concept is the idea that solvolyses proceed via a spectrum of reaction mechanisms. An S_N_1-S_N_2 spectrum of mechanisms is well established for many solvolyses (e.g., solvolyses of secondary alkyl sulfonates [5]). Density functional (DFT) calculations for benzoyl chlorides [6] were consistent with concerted S_N_2 reactions, with “loose” (cationic) transition states for electron-donating substituents and “tight” (associative) transitions states for electron withdrawing substituents (Figure 1). Very recent DFT calculations [7] further illustrated the S_N_1-S_N_2 spectrum (e.g., for benzyl halides), but also provided support for the proposal [8] that there was also an S_N_2-S_N_3 spectrum of mechanisms for solvolyses of aromatic sulfonyl chlorides (**2**).

Electrophilic solvation of the developing chloride anion and general solvation are excluded from the assessment of molecularity, and are not shown in Figure 1. At the S_N_1 extreme of the S_N_1-S_N_2 spectrum, there may be evidence for a cationic intermediate and reactions are unimolecular (e.g., solvolyses of *p*-dimethylaminobenzoyl fluoride (**3**, Z = NMe_2_) show common ion rate depression [9]).

The key feature of the S_N_3 mechanism is that the reactions are termolecular: a solvent nucleophile attacks the substrate (e.g., RX or *p*-Z-substituted benzoyl chlorides as in Figure 1b), and a second solvent molecule assists by partially deprotonating the nucleophile (three molecules undergo covalency change, but they could be preassembled by hydrogen bonding).

Although the kinetic order is usually first order (rate constant *k*_obs_), it has proved useful in some cases to calculate various third order rate constants (*k*_3_); as shown below, values of log *k*_3_ may be approximately constant or may give good linear plots *vs.* solvent polarity parameters for solvolyses of acid derivatives. Furthermore, deviations from linearity provide some of the clearest evidence for the relatively subtle change in mechanism from the end of the S_N_1-S_N_2 spectrum to the beginning of the S_N_2-S_N_3 spectrum.

## 2. Results and Discussion

**Figure 2:**
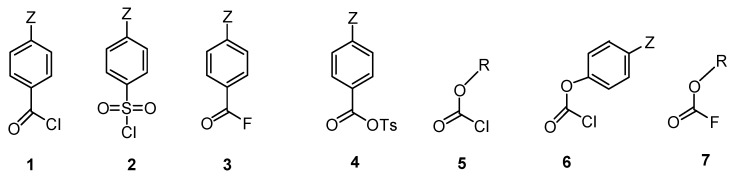
The substrates (**1–7**) are: named as follows: (**1**) *p*-Z-substituted benzoyl chlorides; (**2**) *p*-Z-substitued sulfonyl chlorides; (**3**) *p*-Z-substituted benzoyl fluorides; (**4**) *p*-Z-substituted benzoyl tosylates, where tosylate (OTs) is *p*-toluensulfonate); (**5**) alkyl chloroformates; (**6**) *p*-Z-substituted phenyl chloroformates; (**7**) alkyl fluoroformates.

Initial results were obtained for the range of substrates shown in Figure 2.

### 2.1. Choice of Equation for Correlating Solvolysis Rates

The simplest (but not reliable) equation for obtaining the kinetic order of a pseudo-first order solvolysis is Equation (1), where n is the kinetic order, *k*_n_ is the nth order rate constant and [water] refers to the molar concentration of water in for example a binary mixture such as acetone-water. Taking logarithms, a plot of log *k*_obs_ *vs.* log [water] would have a slope of n (Equation (2)). To illustrate the lack of reliability of Equation (2), two examples using kinetic data from the recent literature (but not the published interpretation of the data) are: (a) solvolyses of benzoyl fluoride (**3**, Z = H) in acetone–water, which would have a kinetic order of 3.3 (kinetic data from Reference [10]); (b) solvolyses of *p*-nitrobenzoyl tosylate (**4**, Z = NO_2_), which would have a kinetic order of 0.3 (kinetic data from Reference [11]).

*k*_obs_ = *k*_n_[water]^n^ (1)

log *k*_obs_ = n × log [water] + log *k*_n_ (2)

An alternative, more credible explanation is that solvolyses of both **3**, Z = H and **4**, Z = OTs are third order. Solvolyses of **3**, Z = H in water and D_2_O give a kinetic solvent isotope effect (KSIE) of 2.0 [9], and the KSIE for **4**, Z = OTs in methanol and MeOD varies from 1.84 at −10 °C to 1.59 at 25 °C [11]. These values are consistent with some cleavage of O-H or O-D bonds in the rate determining step, as shown in Figure 1b. Various reactions of acid derivatives including esters are thought to react by such general base-catalysed processes [12].

There are many publications in which the KSIE for MeOH and MeOD were obtained (see below), and a wide range of KSIE data have been published by Koo *et al.* [13,14]; two of these were included in the recent DFT calculations [7]: values of KSIE in water are: for **2**, Z = OMe (calc. 1.64, expt. 1.37); for **2**, Z = NO_2_ (calc. 1.97, expt. 1.76). Both S_N_1 [15] and S_N_2 [16] reactions typically have KSIEs <1.3 (see also Table 5 of Reference [8]), so hydrolyses of **2**, Z = OMe are close to those expected for S_N_2 reactions, but those for **2**, Z = NO_2_ are significantly higher.

**Figure 3:**
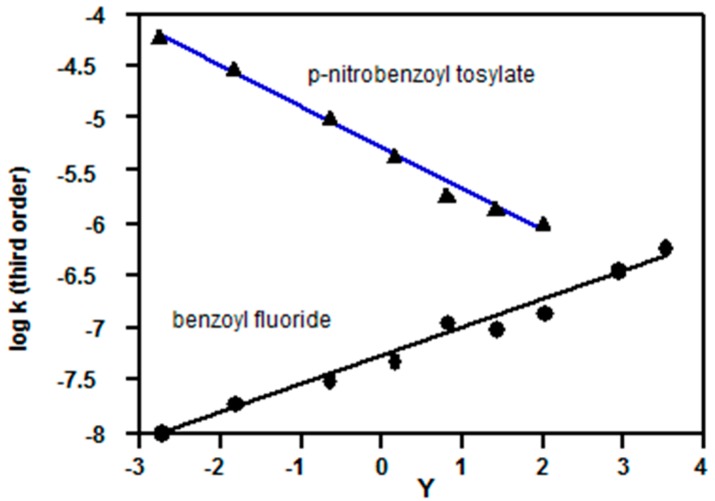
Plots of logarithms of third order rate constants (Equation (3)) *vs.* Grunwald-Winstein *Y* values [4,17] for acetone-water mixtures; kinetic data for benzoyl fluoride (3, Z = H) at 25 °C are from Reference [10]; kinetic data for *p*-nitrobenzoyl tosylate (4, Z = NO_2_) at −10 °C are from Reference [11].

Calculated third order rate constants Equation (3) give satisfactory plots *vs.* the Grunwald-Winstein (GW) parameter (*Y*) for solvent ionizing power [4,17,18] (a measure of solvent polarity). The plots (Figure 3) show that calculated third order rate constants for solvolyses of benzoyl fluoride (**3**, Z = H) increase as *Y* increases (*slope* = 0.27 ± 0.02, *R*^2^ = 0.979, std. error = 0.09, *n* = 9), but those for *p*-nitrobenzoyl tosylate (**4**, Z = NO_2_) decrease (*slope* = −0.40 ± 0.02, *R*^2^ = 0.994, std. error = 0.06, *n* = 7).

log *k*_3_ = log {*k*_obs_/[water]^2^} = log *k*_obs_ − 2 log [water] = *slope* × *Y* + *intercept* (3)

It should not be inferred that the linearity of the plots (Figure 3) is by itself evidence for third order reactions, because a plot (not shown) of log [water]^2^ *vs.* *Y* is close to linear, but curves downwards for 80% and 90% acetone-water; this curvature can lead to a “kink” in Equation (3) plots, Consequently, the main difference between Equation (3) and a GW plot of log *k*_obs_ *vs.* *Y* is that the slopes of Equation (3) are lower, but it some cases (see below) mechanistic changes are indicated much more clearly using Equation (3); note however that for bimolecular reactions in 80% and 90% acetone-water, there may be a change in slope which is not due to a mechanistic change.

As with other mechanistic indicators, it is always desirable to provide independent evidence, and KSIE data will be quoted extensively during the following discussion. Multi-parameter equations provide a more comprehensive account of solvent effects, and these are discussed in Section 2.4.

Also, there are various examples of chloroformates (**5**,**6**) where the *slope* based on Equation (3) is close to zero, implying that a plot of log *k*_obs_ *vs.* log [H_2_O] (Equation (2)) would give a value of n (the kinetic order with respect to water) of close to 2 (e.g., see Section 2.2.1).

An important advantage of Equation (3) over Equation (2) is that trends in slopes can be interpreted with the benefit of long experience of applications of the original GW equation [18]. The selection of *Y* rather than one of the other *Y*_X_ parameters [19] for solvent ionizing power ensures that none of the *Y* values are based on extrapolated data, and that slopes for all substrates are comparable (for further discussion on this choice, see Reference [20]). For many aqueous binary mixtures (including acetone-water), to a good approximation *Y* = *Y*_Cl_ × 0.75 (see Table 8 of Reference [19]). The factors influencing the slopes of correlations based on Equation (3) were investigated for a wide range of substrates.

### 2.2. Evidence for the Third Order (Addition-Elimination) Reaction Channel from Correlations Using Equation (3) and Kinetic Solvent Isotope Effects

#### 2.2.1. Chloroformates

In general, chloroformates (**5,6**) are much more likely than the corresponding carboxylic acid chlorides (**2**) to react via higher order (associative) reactions. Recent G3 calculations of gas phase heterolytic bond dissociation energies (HBDE) for the C–Cl bonds show for example that PhCOCl (**1**, Z = H) has an HBDE of 150.1 kcal/mol whereas the corresponding chloroformate (**6**, Z = H) has a much higher HBDE of 166.5 kcal/mol [21]. Consequently, the electrophilic “pull” is less for chloroformates, and a greater nucleophilic “push” might be expected [22].

**Figure 4:**
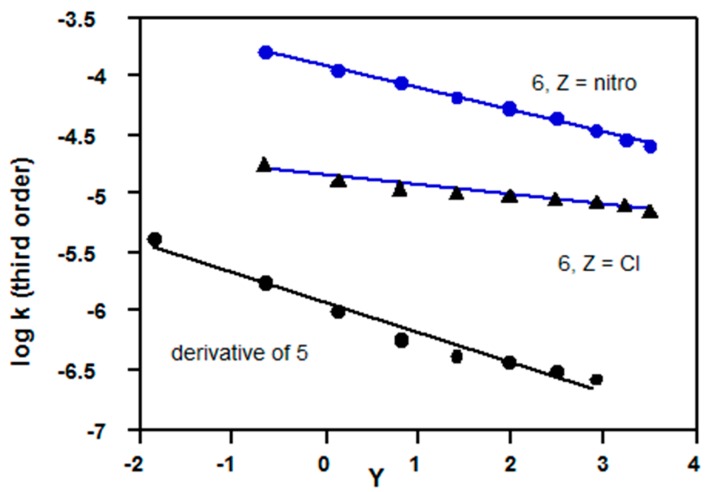
Plots of logarithms of third order rate constants (Equation (3)) *vs.* Grunwald-Winstein *Y* values [4,17] for acetone-water mixtures; kinetic data: for **5**, R = C(Me)_2_CCl_3_ at 25 °C from Reference [26]; for *p*-chlorophenyl chloroformate (**6**, Z = Cl) at 25 °C from Reference [14]; for *p*-nitrophenyl chloroformate (**6**, Z = NO_2_) at 25 °C from Reference [27].

Inspection of each column of Table 1, shows that logarithms of the calculated third order rate constants (log *k*_3_) are almost independent of solvent composition for solvolyses ethyl chloroformate (**5**, R = Et), phenyl- and substituted phenyl chloroformates (**6**, Z = H, Me, OMe), and trichloroethyl chloroformate (**5**, R = CH_2_CCl_3_) in a wide range of acetone-water mixtures. Equation (3) gives slightly negative slopes for solvolyses of: (**5**, R = C(Me)_2_CCl_3_) for which *slope* = −0.26 ± 0.02, *R*^2^ = 0.969, std. error = 0.08); **6**, Z = Cl (*slope* = −0.08 ± 0.01, *R*^2^ = 0.946, std. error = 0.03) and **6**, Z = NO_2_ (*slope* = −0.19 ± 0.01, *R*^2^ = 0.994, std. error = 0.02). All three are plots may alternatively be interpreted as slightly curved (Figure 4).

**Table 1:** Approximately constant values of logarithms of calculated third order rate constants (Equation (3)) for solvolyses of chloroformates in acetone-water.

Acetone (% *v*/*v*)	5, R = Et *^a^* 24.2 °C	6, Z = H *^b^* 25 °C	6, Z = Me *^b^* 25 °C	6, Z = OMe *^b^* 25 °C	5, R = CH_2_CCl_3_ Reference *^c^*, 35 °C
**95**	−7.01				
**90**	−7.01	−5.11 *^d^*			−4.62
**80**	−7.11	−5.20	−5.43	−5.47	−4.82
**70**		−5.35	−5.54	−5.55	−4.83
**60**	−7.13	−5.40	−5.59	−5.60	−4.86
**50**		−5.43	−5.59	−5.58	−4.82
**40**	−7.06	−5.41	−5.56	−5.53	−4.78
**30**		−5.36	−5.51	−5.47	−4.67
**20**	−6.96	−5.34	−5.46	−5.43	−4.62
**10**		−5.34	−5.45	−5.39	
**water**	−6.96	−5.37	−5.46	−5.55	

*^a^* Data from Reference [23]; *^b^* Data from Reference [14], unless stated otherwise; *^c^* Data from Reference [24]; *^d^* Data from Reference [25].

Data for solvolyses of many other chloroformates [28] in a limited range of acetone-water solvent compositions, usually within the range 90%–60% show the same trends as those discussed above. Exceptions to these trends indicate the possibility of mechanistic changes, as discussed in Section 2.3.

#### 2.2.2. Fluoroformates (7)

One of the first extensive study of solvent effects on these solvolyses was for *n*-octyl fluoroformate (7, R = *n*-octyl); data in 95%, 90%, 80% and 60% *v*/*v* acetone-water [29] give a positive *slope* based on Equation (3) of 0.22 ± 0.02. Positive *slopes* are observed for many other fluoroformates, as well as for benzoyl fluoride (**3**, Z = H)—see Figure 3. Details are not given here because of the limited range of acetone-water solvent compositions (usually between 90% and 60% *v*/*v* [28]). Instead, typical studies involve a much wider range of pure solvents and binary solvent mixtures (Section 2.4), and mechanistic assignments are supported by studies of KSIEs in MeOH [28].

Usually solvolyses of fluoroformates in MeOH give KSIEs >2, consistent with general base catalysis and the mechanism shown in Figure 1b. However, *t*-butyl fluoroformate (7, R = Bu*^t^*) gives a KSIE in MeOH of 1.26 [30], characteristic of the cationic reaction channel (Figure 1a). Also based on Equation (3), the *slope* for 7, R = Bu*^t^* is 0.33 ± 0.02 in 90%, 80%, 70% and 60% acetone-water. Consequently, even if a wider range of data was available, changes in mechanism may not lead to convincing changes in slopes for solvolyses of fluoroformates in acetone-water mixtures. In contrast, carboxylic acid and sulfonyl chlorides do show clear changes in slopes (see Section 3).

When a wide range of solvents of varying ionizing power and nucleophilicity are investigated, it is possible to separate the two mechanisms; the extended Grunwald-Winstein equation (Section 2.4) can then be used to correlate the kinetic data and to obtain *m* values for each mechanism are obtained. It was found that the slopes (*m* values) for the cationic channel were lower than for the associative channel; both channels were observed for 1-adamantyl fluoroformates [31], and the ionization channel for *t*-butyl fluoroformate gave an *m* value of 0.41 [30], slightly lower than typical values for the associative channel (see Table 4 of Reference [28]). This is the opposite trend to that observed for chloroformates [32] and other acid chlorides (see below).

#### 2.2.3. Carboxylic Acid Halides

Solvolyses of many carboxylic acid chlorides in a single binary solvent mixture (such as acetone-water) would give linear plots of log *k*_obs_ or log *k*_3_ *vs.* *Y*. Examples discussed in this section will be restricted to those for which KSIE data indicates the possibility of an S_N_3 mechanism.

Good linear correlations of log *k*_3_ *vs.* *Y* (Equation (3)) for 90%–20% acetone-water have already been published for solvolyses of *p*-nitrobenzoyl chloride (**1**, Z = NO_2_) and chloroacetyl chloride (ClCH_2_COCl): *slope* = −0.18, see Figure 2 of Reference [33] and Figure 3 of Reference [34] respectively. In each case the KSIE in MeOH is >2. Extrapolation using equation (4) of the 90%–20% acetone plot [33] to pure water gives a predicted value of *k* = 0.068 ± 0.004, in satisfactory agreement with the experimentally observed values of *k*_obs_ = 0.062 [9] and 0.056 [35] published subsequently. Also the KSIE in water is approximately 2, although published values vary from 1.75 to 2.3 [35].

log *k*_3_ (for **1**, Z = NO_2_) = −0.176*Y* − 4.046 (4)

**Figure 5:**
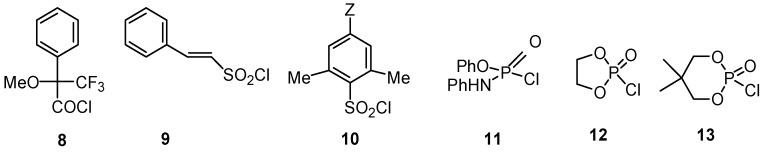
Structures of additional substrates, named as follows: α-Methoxy-α-(trifluoromethyl) phenylacetylchloride (**8**); *trans*-β-styrenylsulfonyl chloride (**9**); 4-substituted-2,6-dimethylbenzene-sulfonylchlorides (**10**); Phenyl-*N*-phenylphosphor-amidochloridate (**11**); 2-chloro-1,3,2-dioxaphospholane-2-oxide (**12**); 2-chloro-5,5-dimethyl-1,3,2-dioxaphosphorinane-2-oxide (**13**).

More highly chlorinated substrates such as CHCl_2_COCl would also be expected to fit the associative mechanism, but experimental data are limited by their high reactivity; this is overcome in the derivative (**8**, Figure 5), deactivated by the substituents [36]; values of log *k*_3_ are approximately constant from 80% acetone to water (the slope of a plot based on Equation (3) is 0.08 ± 0.03), and also *k*_MeOH_/*k*_MeOD_ = 2.4 [36].

Solvolyses of **1**, Z = NO_2_ from 90% acetone to water [33] and of **8** from 80% acetone to water [36] fit all of the suggested criteria for a third order associative mechanism (*i.e*., KSIE in MeOH and water >1.5, and a linear plot Equation (3), characteristic of the third order reaction channel (Figure 1b). Also, logarithms of observed rate constants for solvolyses of **1**, Z = NO_2_ and methyl chloroformate (MeOCOCl) correlate linearly with those for chloroacetyl chloride (see Figure 1 of Reference [34]), showing strong similarities between solvolyses of chloroformates and other acid chlorides.

Based on the observations [9,10] for benzoyl fluoride (Figure 3), and the reduced “pull” of fluoride as a leaving group, it would be expected that solvolyses of typical acid fluorides would react via the third order mechanism. Supporting evidence includes: (i) the solvent effect, *k* (water)/*k* (50% acetone-water), of 14 for spontaneous hydrolyses of acetyl fluoride can be compared with that of 22.6 [10] for benzoyl fluoride—hydrolyses are also acid and base-catalysed [37]; (ii) observed rates of solvolyses of *p*-dimethylaminobenzoyl fluoride (**3**, Z = NMe_2_) are almost independent of solvent composition from 80% to 60% ethanol, before a mechanistic change occurs to a cationic pathway [9].

#### 2.2.4. Sulfonyl Chlorides

Sulfonyl chorides have relatively high heterolytic bond dissociation energies [21], implying that formation of sulfonyl cations is unfavourable. Even when there is an adjacent nitrogen lone pair, as in Me_2_NSO_2_Cl, solvolyses do not show common-ion effects [38]. Comparison with reactions of various sulfonyl chlorides in acetone-water by plotting log *k*_obs_*vs. Y* gave a relatively high value of *m* = 0.69 for Me_2_NSO_2_Cl, leading to the conclusion that the transition state was more ionic (looser) than for other sulfonyl chlorides, but the mechanism was not S_N_1 as suggested by others [39]; the KSIE in water and D_2_O of 1.3 (Table 2, References [38,39,40,41,42,43,44,45]) is compatible with either S_N_1 or S_N_2 mechanisms. In the terminology of Figure 1, the mechanism of solvolysis of Me_2_NSO_2_Cl, is S_N_2, and within the S_N_2-S_N_1 spectrum throughout the wide range of *Y* values, but is not S_N_1 even in water. Recent more extensive studies of solvent effects also led to the proposal of an S_N_2 mechanism [46].

**Table 2:** Correlations Equation (3) for acetone-water (% *v*/*v*) and kinetic solvent isotope effects (KSIE) in water and methanol for solvolyses of sulfonyl chlorides at 25 °C (unless stated otherwise).

Substrate	Slope	Intercept	*R*^2^	Std. Error	Range (%)	KSIE *^a^*	KSIE *^b^*
**Me_2_NSO_2_Cl *^c^***	0.36 ± 0.01	−7.16 ± 0.03	0.990	0.06	80–0	1.33 *^c^*,1.29 *^d^*	
**9 *^e^*^,*f*^**	0.16 ± 0.02	−6.05 ± 0.04	0.882	0.11	90–0 *^g^*	1.46	1.76
***i*-PrSO_2_Cl *^h^***	0.07 ± 0.02	−8.18 ± 0.03	0.770	0.06	80–0	1.66	2.54
**MeSO_2_Cl *^i^***	−0.07 ± 0.02	−6.81 ± 0.03	0.788	0.06	80–0	1.81	1.62
**PhCH_2_SO_2_Cl *^j^***	−0.07 ± 0.02	−6.25 ± 0.04	0.688	0.08	90–20		2.34
**2, Z = NO_2_*^k^***	−0.11 ± 0.01	−5.56 ± 0.03	0.922	0.06	90–0	1.76 *^l^*	2.31 *^l^*
**2, Z = Cl *^m^***	0.04 ± 0.02	−6.32 ± 0.03	0.461	0.07	90–0	1.65 *^l^*	1.89 *^l^*
**2, Z = H *^m^***	0.13 ± 0.03	−6.56 ± 0.06	0.781	0.13	90–0	1.59 *^l^*	1.79 *^l^*
**CF_3_SO_2_Cl *^e^*^,*n*^**	−0.42 ± 0.03	−6.14 ± 0.06	0.971	0.11	80–0	2.24	3.08

^a^ Refers to *k*_water_/*k*_D2O_; *^b^* Refers to *k*_MeOH_/*k*_MeOD_; *^c^* Reference [38]; *^d^* Reference [39]; *^e^* At 45 °C; *^f^* Reference [40]; *^g^* If the data point for 90% acetone-water is omitted: slope = 0.20 ± 0.02; intercept = −6.14 ± 0.04; *R*^2^ = 0.953; std. error = 0.07; *^h^* Reference [41]; *^i^* Reference [42]; *^j^* At 35 °C, Reference [43]; *^k^* Reference [44]; *^l^* Reference [13]; *^m^* Reference [8]; *^n^* Reference [45], including curved plots of log *k*_obs_ *vs.* *Y*_Cl_ and linear plots of log *k*_3_ *vs.* *Y*_Cl_.

Doubts about the interpretation of KSIE data in terms of general base catalysis were based [40,47] on the anomalously high value (2.54) for the KSIE of *i*-PrSO_2_Cl in MeOH (Table 2). Also the low value of 1.62 for MeSO_2_Cl does not fit the general trend. These outliers obscure the general trend of higher values for KSIE and very low and/or negative Equation (3) slopes for typical sulfonyl chlorides (Table 2). Apart from the first and perhaps second entries in Table 2, the slopes and particularly the KSIE values of the remaining substrates are consistent with mechanistic changes within the S_N_2-S_N_3 spectrum (Figure 1b) throughout the range of acetone-water mixtures. As the concentration of acetone increases, ΔS^≠^ becomes progressively more negative for solvolyses of **2**, Z = NO_2_, Br, H and Me [48,49], indicating more highly ordered transition states.

Most of the slopes shown in Table 2 are so low that the correlation with *Y* is poor. Typical plots have a very shallow U-shape. Arenesulfonyl chlorides (**2**,**10**) permit the systematic introduction of more electron donating groups, and U-shaped curvature of the Equation (3) plots becomes progressively more pronounced. These results are discussed in Section 2.3.3.

#### 2.2.5. Anhydrides

Data [11] for solvolyses of *p*-nitrobenzoyl tosylate (**4**, Z = NO_2_) show a linear plot (Figure 3) based on Equation (3) and a KSIE of *ca*. 1.7 in methanol [11], consistent with a third order mechanism (Section 2.1). Other anhydrides show lower KSIE values: e.g., in methanol for acetyl tosylate (CH_3_COOTs, KSIE = 0.99 at −39.6 °C [50]), benzoyl tosylate (PhCOOTs, KSIE = 1.1 at −10 °C [11]), and other anhydrides (PhSO_2_)_2_O, Ts_2_O, Ms_2_O) KSIE = 1.35–1.4 at −10 °C [51,52]); in mixed organic/water mixtures of 1.2–1.3 for solvolyses of various aromatic sulfonic acid anhydrides at 22.5 °C [53]. These results are consistent with changes within the S_N_2-S_N_1 spectrum. A rationalization is that the electrophilic “pull”of sulfonates is much larger than for chlorides (e.g., *k*_OMs_/*k*_Cl_ ~ 10^3^ for methanesulfonyl derivatives [52]), so the extra “push” of a third order process is not required.

For solvolyses *p*-bromophenylsulfonic acid anhydride in acetone-water mixtures, an Equation (2) slope of 1.75 was reported [54]. However, these data do not provide sufficient support for a third order mechanism, because of the unreliability of Equation (2)—see Section 2.1. Slopes of plots *vs.* *Y* depend on the size of the leaving group, probably due to the extent of charge delocalisation in the developing anion; for S_N_1 solvolyses of 1-adamantyl substrates an order of slopes Cl > Br > I > OTs was established [55]; tosylates have relatively low slopes, and given the trend, it would be expected that fluorides would have the highest slopes (e.g., Figure 3).

#### 2.2.6. Phosphorus Halides

KSIE data are few (Table 3, References [56,57,58,59,60,61,62,63]), although there is an additional value of 1.25 for (Pr^i^O)_2_POCl in *k*_water_/*k*_D2O_ [64]. (PhO)_2_POCl shows approximately constant values of log *k*_3_ and KSIE of **3**, consistent with third order kinetics and general base catalysis by a second water molecule [61]; Hartree-Fock and DFT levels calculations [65] for (RO)_2_POX (R = H or Me, X = F or Cl) supported this proposal [65]. Other substrates show low and usually positive slopes, due to small changes in log *k*_3_, and hence a poor correlation with *Y*. The largest positive slope is 0.28 for Ph_2_PSCl, but the data refer only to 95%–50% acetone-water [63]. Emphasising the importance of nucleophilic attack, it is usually stated that the hydrolyses are bimolecular [57,58,59,60,61,62,63,64,65]; it is now suggested that additional KSIE data would confirm the role of catalysis by a second solvent molecule.

**Table 3:** Results (Equation (3)) for acetone-water (% *v*/*v*) and kinetic solvent isotope effects (KSIE) in water and methanol for solvolyses of phosphorus chlorides at 25 °C (unless stated otherwise).

Substrate	Slope	Intercept	*R*^2^	Std. Error	Range (%)	KSIE *^a^*	KSIE *^b^*
**(Me_2_N)_2_POCl *^c^***	0.17 ± 0.03	−6.28 ± 0.06	0.930	0.09	80–0	1.34 *^d^*	
**11 *^e^***	0.15 ± 0.06 *^f^*	−6.55 ± 0.12	0.463	0.30	90–0		
**(MeO)_2_POCl *^g^***	0.07 ± 0.01	−5.08 ± 0.01	0.979	0.02	90–60		
**12 *^h^***	0.16 ± 0.03	−4.22 ± 0.04	0.884	0.10	90–20		
**13 *^h^*^,*i*^**	−0.06 ± 0.02	−6.22 ± 0.04	0.597	0.08	80–0		
**(PhO)_2_POCl *^j^***	0 *^k^*				95–0	2.9 *^l^*	3.06
**Ph_2_POCl *^m^***	0 *^n^*				95–50		
**Ph_2_PSCl *^o^***	0.28 ± 0.03	−4.90 ± 0.04	0.967	0.09	95–50		1.83
**(MeO)_2_PSCl *^g^***	0.08 ± 0.03	−6.67 ± 0.06	0.503	0.15	90–0		

^a^ Refers to *k*_water_/*k*_D2O_; *^b^* Refers to *k*_MeOH_/*k*_MeOD_; *^c^* Reference [56]; *^d^* Data from Reference [57] at 10 °C; *^e^* Reference [58]; *^f^* Shallow-U-shaped plot; *^g^* Reference [59]; *^h^* Reference [60]; *^i^* At 50 °C; *^j^* Reference [61]; *^k^* Very shallow U-shaped plot, with all values of log *k*_3_ within the narrow range −4.8 to −5.26; *^l^* At 5 °C; *^m^* Reference [62]; *^n^* Values of log *k*_3_ vary within a very narrow range (−2.87 to −2.97); *^o^* Data from Reference [63] at 55 °C.

### 2.3. Deviations from Equation (3) as Evidence for Mechanistic Changes

#### 2.3.1. Carboxylic Acid Chlorides

**Figure 6:**
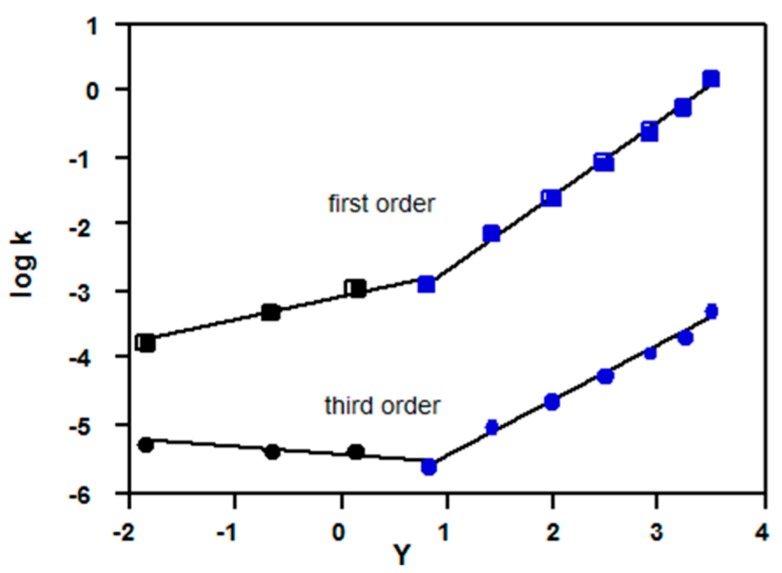
Comparison of plots of logarithms of first order rate constants (log *k*_obs_) and third order rate constants (log *k*_3_, Equation (3)) *vs.* Grunwald-Winstein *Y* values [4,17] for solvolyses of benzoyl chloride (**1**, Z = H) in acetone-water mixtures at 25 °C; kinetic data from Reference [68].

From the results given in Section 2.2.3 (supported by the more extensive data for chloroformates in Section 2.2.1), the normal expectation is that third order solvolyses in acetone-water will fit Equation (3) with zero or negative slopes (mechanism shown in Figure 1b). In contrast, when the cationic mechanism (Figure 1a) operates, there is a much higher and positive slope (e.g., see Figure 1 of Reference [66]). Consequently, it is possible to detect a change in mechanism from third order to cationic by plotting log *k*_obs_ *vs.* *Y*; e.g., for solvolyses of benzoyl chloride (**1**, Z = H) in a wide range of acetone-water mixtures [67]. In addition, a much clearer change in slopes is obtained by plotting log *k*_3_*vs. Y* (Equation (3)) instead of log *k*_obs_; the two plots are compared in Figure 6.

Close to the point where the two lines intersect (Figure 6, around *Y* = 1, ~60% acetone-water), the two mechanisms are assumed to be concurrent, and so values of log *k*_obs_ and log *k*_3_ are corrected by subtracting log 2 = 0.3 from both *Y* = 1 values (these points are then fitted to both relevant correlation lines).

Solvolyses of *p*-chlorobenzoyl chloride (**1**, Z = Cl) and phenylacetyl chloride in acetone water also show sharp changes in slope when log *k*_3_ is plotted *vs.* *Y* for acetone-water mixtures (see Figure 4 of Reference [69] and Figure 3 of Reference [34]). In contrast, the same plots for *p*-nitrobenzoyl chloride (**1**, Z = NO_2_) and chloroacetyl chloride are linear throughout the range of acetone-water mixtures (see Section 2.2.3). Solvolyses of trimethylacetyl chloride give a similar plot to Figure 6 when log *k*_obs_ is plotted *vs. Y* (see Figure 2 of Reference [70]). For solvolyses of *p*-dimethylaminobenzoyl fluoride (3, Z = NMe_2_) in ethanol-water mixtures, a sharp change in slope is readily apparent even in plots [9] of first order rate constants (log *k*_obs_).

The above results are significant because they show that a change in hydrolysis mechanism can occur when the % water in a binary mixture such as acetone-water is altered.

#### 2.3.2. Chloroformates

**Figure 7:**
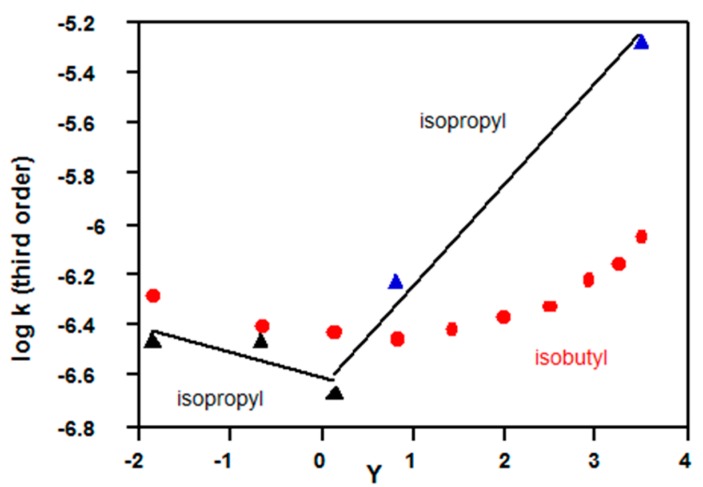
Changes in mechanism based on plots of log *k*_3_ *vs. Y* Equation (3) for solvolyses in acetone-water of **5**, R = *^i^*Pr at 40 °C and **5**, R = CH_2_CH(CH_3_)_2_ at 45 °C; kinetic data from References [71,72,73].

Solvolyses of isopropyl chloroformate (**5**, R = *^i^*Pr) show a KSIE in water at 25 °C of only 1.25 [71], in contrast to values of 1.8–2.2 for methyl, ethyl and aryl derivatives [72]; also, values of log *k*_3_ in water for **5**, R = *^i^*Pr deviate substantially from the downward trend set by 90%–70% acetone-water compositions (Figure 7). These results are in marked contrast to solvolyses of ethyl chloroformate (**5**, R = Et), which shows a close to constant value of log *k*_3_ from 95% *v*/*v* acetone-water to 100% water (Table 1). It can be concluded that both **5**, R = Et and **5**, R = *^i^*Pr are hydrolysed by the third order mechanism (Figure 1b) in 90% and 80% acetone-water mixtures, but the mechanism changes to cationic for hydrolysis of **5**, R = *^i^*Pr in water. The minimum at *Y*~0 corresponds to 70% acetone, and log 2 = 0.3 was subtracted from this data point before fitting it to both correlation lines (Figure 7).

The above conclusion is in good agreement with published work [72], using an independent approach. From the extended GW equation (Section 2.4) for only seven solvents clearly favouring the cationic mechanism (water, formic acid, and various CF_3_CH_2_OH-water and (CF_3_)_2_CHOH-water mixtures), it was calculated [72] that the mechanistic changeover for 5, R = *^i^*Pr occurred between 70 and 80% acetone-water. The prediction in Figure 7 that the mechanistic change occurs in 70% acetone-water is based on deviations from an independent correlation for the third order mechanism.

The plot (red in Figure 7) of log *k*_3_ *vs. Y* for solvolyses of isobutyl chloroformate (**5**, R = CH_2_CH(CH_3_)_2_) show very similar values to those for **5**, R = *^i^*Pr in 90%–60% acetone-water (first 4 data points); note that the temperatures are not identical (40 and 45 °C). Compared with the line drawn for **5**, R = *^i^*Pr, additional data in 50%–10% acetone for **5**, R = CH_2_CH(CH_3_)_2_ show a smaller change in slope; also the KSIE in water of 1.54 [73] is larger. The KSIE in methanol of 2.03 [73] and the low slope of the plot for 90%–60% acetone (*Y* values from −2 to 1) are consistent with third order reactions, and a change to a bimolecular mechanism was suggested for reaction in water [73]. Comparing slopes and KSIE values in water, it appears that the change in mechanism begins slightly later and is more gradual for **5**, R = CH_2_CH(CH_3_)_2_ than for **5**, R = *^i^*Pr.

**Figure 8:**
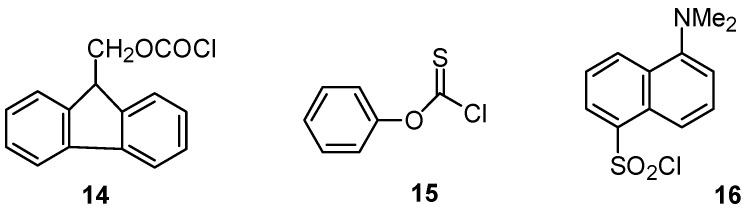
Structures of additional substrates: 9-fluorenylmethyl chloroformate (**14**); phenyl chlorothionoformate (**15**); 5-dimethylamino-naphthalene-1-sulfonyl (dansyl) chloride (**16**).

Both 9-fluorenylmethyl chloroformate (**14**, Figure 8) and **5**, R = CH_2_CH(CH_3_)_2_ have a β,β-dialkyl group and solvolyses of **14** in acetone-water [74] also give shallow U-shaped plot (not shown); values of log *k*_3_ at 45 °C vary from −5.75 (90% acetone) to −6.11 (50% acetone) and then up to −5.70 (20% acetone); the KSIE in methanol is 2.2, but there are no KSIE data for water [74].

**Figure 9:**
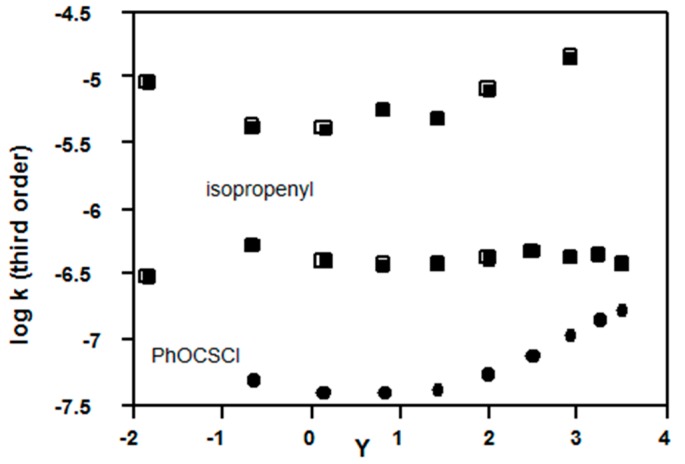
Plots of log *k*_3_ *vs. Y* (Equation (3)) for solvolyses in acetone-water of phenylthiono-formate (**15**) at 25 °C, and isopropenyl chloroformate (**5**, R = CH_3_C=CH_2_) at 35 (upper plot) and 10 °C (middle plot); kinetic data from References [75,76,77].

An almost identical pattern of results is observed (Figure 9) for solvolyses of phenyl chlorothionoformate (**15**); log *k*_3_ values are close to constant from *Y* = −1 to +2 (80%–40% acetone); then there is an increase in slope (Figure 9); the KSIE value are 2.02 for methanol, but only 1.45 for water [75], so a gradual change to a bimolecular mechanism may be underway.

Equation (3) plots for isopropenyl chloroformate (**5**, R = CH_3_C=CH_2_) at 10 °C show close to constant values of log *k*_3_ from 90% acetone to water (Figure 9, middle), whereas the upper plot at 35 °C is U-shaped. Solvent effects and KSIE values in water and methanol of >2.0 at 10 °C are very similar to those for **1**, Z = NO_2_ [76], so there is strong evidence for the third order mechanism in acetone-water. At 35 °C [77], there may be an increase in slope from 50% to 20% acetone-water (*Y* > 1.4, Figure 9) and a bimolecular cationic mechanism (Figure 1a) having a higher activation energy may start to be competitive. Evidence for a change to the cationic reaction mechanism was later obtained by comparing solvolyses in fluorinated alcohols with those for phenyl chloroformate [78].

#### 2.3.3. Sulfonyl Chlorides

**Figure 10:**
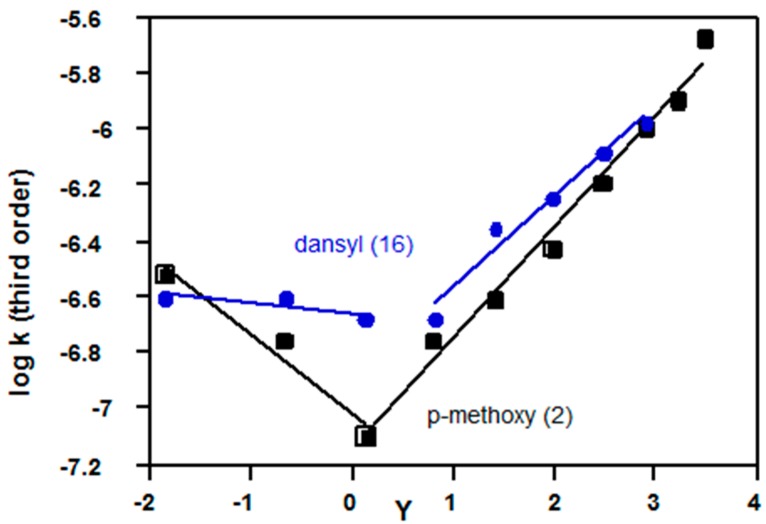
Plots of log *k*_3_ *vs. Y* (Equation (3)) for solvolyses in acetone-water of 4-methoxybenzene sulfonyl (**2**, Z = OMe) at 25 °C, and dansyl chlorides (**16**) at 35 °C; data from References [79,81,82].

**Figure 11:**
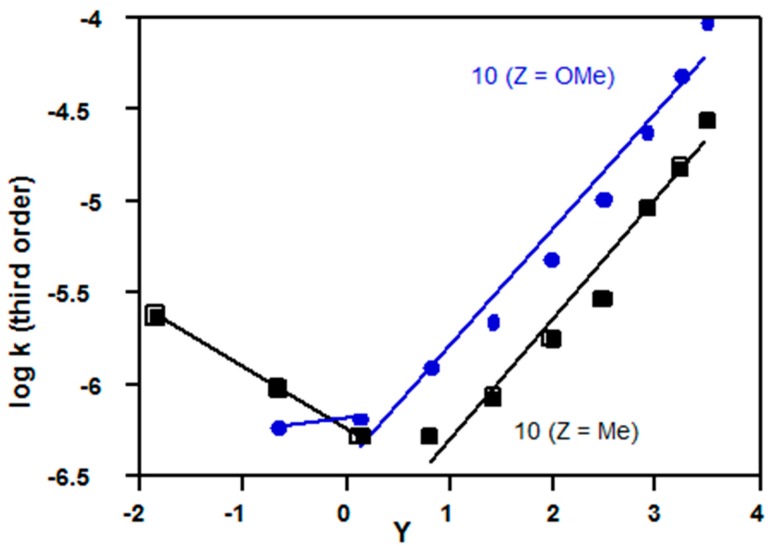
Plots of log *k*_3_ *vs. Y* Equation (3) for solvolyses in acetone-water of 4-*Z*-2,6-di-methylbenzene sulfonyl chloride (**10**, Z = Me, OMe) at 25 °C; kinetic data from References [80,81].

Although most sulfonyl chlorides react by the third order mechanism within the S_N_3-S_N_2 spectrum (Section 2.2.4), there is evidence for a mechanistic changeover to the S_N_2-S_N_1 spectrum for electron-rich substrates; however, formation of sulfonyl cations is relatively unfavourable [21], and there is no evidence for an S_N_1 mechanism. Evidence for the changeover will be based on KSIE data (Table 4, References [79,80,81]) and non-linear Equation (3) plots (Figure 10 and Figure 11), but there is also support from independent evidence on alcohol/water selectivities [79,81,82].

In all four examples (Table 4), the KSIE decreases from >1.5 in methanol (S_N_3-S_N_2 spectrum) to reach a value of 1.3–1.4 more characteristic of the S_N_2-S_N_1 spectrum. Also, the substituent effect for 4-substituted 2,6-dimethyl substrates (**10**) changes from *k*_OMe_ < *k*_Me_ on the left side of Figure 11 (S_N_3-S_N_2 spectrum) to *k*_OMe_ > *k*_Me_ on the right side (consistent with relatively more bond breaking for the S_N_2-S_N_1 spectrum).

**Table 4:** Mechanistic changeover for solvolyses of electron-rich sulfonyl chlorides.

Sulfonyl Chloride	KSIE (MeOH)	KSIE (Water) *^a^*	Equation (3) Plot
***p*-methoxybenzene (2, Z = OMe)**	1.58 *^a^*	1.37 *^a^*	Figure 10
**Dansyl (16)**	1.88 *^b^*	(1.34) *^b^*^,*c*^	Figure 10
**Mesityl (10, Z = Me)**	1.68 *^d^*	1.34 *^d^*	Figure 11
***p*-methoxy (10, Z = OMe)**	1.58 *^e^*	1.41 *^a^*	Figure 11

*^a^* Data from Reference [13] at 25 °C; *^b^* Reference [79] at 35 °C; *^c^* Refers to 50/50 MeOH/water; *^d^* Reference [80] at 25 °C; *^e^* Reference [81] at 25 °C.

For solvolyses of **2**, Z = OMe, the changes in slopes based on Equation (3) are large, and the minimum value of log *k*_3_ calculated from experimental data was lowered by log 2 = 0.3, before fitting the two correlation lines (Figure 10).

For solvolyses of **10**, Z = Me (Figure 11), and **16** (Figure 10), there are two minimum values of log *k*_3_ between *Y* = 0 and *Y* = 1; each of these was lowered by 0.15 before fitting the two correlation lines; for solvolyses of **10**, Z = OMe, there is no clear minimum, so lines are drawn through the unadjusted values of log *k*_3_ (coded in blue, Figure 11).

### 2.4. Multi-Parameter Equations

Most of the above discussion has been concerned with solvolyses in water, acetone-water and methanol. Correlations of kinetic data for a wider range of solvents require multi-parameter correlations. In theory these are more comprehensive, but they are also more complex, and caution is recommended [83].

Retaining the assumption of third order rate laws and water as one of the solvents, the possibility of cosolvent acting as general base and/or as nucleophile leads to additional third order rate constants. Assuming that the cosolvent (e.g., acetone, acetonitrile, dioxan) acts as general base (or in some other way), but not as nucleophile, two third order rate constants (*k*_ww_ and *k*_wc_) contribute to *k*_obs_, and Equation (5) is obtained [35,44].

*k*_obs_/[water]^2^ = *k*_ww_ + *k*_wc_ [cosolvent]/[water] (5)

If the cosolvent is an alcohol, it could act as nucleophile and/or general base, and four third order rate constants are then possible. In such cases there are two products, and product ratios provide useful additional information [35,44].

A more general approach, applicable to a very wide range of solvents and solvent mixtures, is to extend the Grunwald-Winstein equation to include a term for solvent nucleophilicity. In this Equation (6), logarithms of observed first order rate constants in any solvent (log *k*), relative to solvolyses in 80% ethanol-water (*k*_o_), are correlated with *Y* and a term (*N*) for solvent nucleophilicity [5]; *m* is the response to changes in *Y* and *l* is the response to changes in *N* and *c* is a residual term.

*log* (*k/k*_o_) = *lN* + *mY* + *c* (6)

Equation (6) was first applied to the S_N_2-S_N_1 spectrum of solvolyses of primary and secondary alkyl tosylates [5]. Solvolyses closer to the S_N_1 extreme have relatively high values of *m* and low values of *l*; as the substrates become more susceptible to nucleophilic attack (e.g., 2-propyl, ethyl, methyl tosylates), values of *m* decrease and values of *l* increase in accordance with Equation (7). In terms of mechanism, a higher *m* implies more electrophilic “pull”, and a higher *l* implies greater nucleophilic “push”; a further implication of Equation (7) is that if there is more “pull” then less push is required (and *vice versa*).

*l=* (1 − *m*)/0.7 (7)

For many of the studies of substrates discussed above, it has become standard practice to interpret *l*/*m* ratios. For an S_N_1 reaction, *l* = 0, so *l*/*m* = 0. As *l* increases and *m* decreases, *l*/*m* ratios >2 can be obtained [40]. Consequently, *l* values and/or *l*/*m* ratios help to position substrates within a spectrum of mechanisms (e.g., for sulfonyl chlorides [40]). Values of *m* depend on how the *N* scale is defined [84], and published values are usually based on the *N*_T_ scale [85,86].

Deviations from a single correlation can be interpreted as evidence for a second reaction channel, and a correlation based on Equation (6) can often be obtained for each reaction channel (e.g., for solvolyses of a variety of ROCOCl, RSCOCl, ROCSCl, and RSCSCl substrates [87]). The two channels can be compared or characterized using *l* and *m* values or the *l*/*m* ratios.

### 2.5. Mechanistic Insights from Theoretical Calculations

Mechanistic conclusions from kinetic studies of hydrolyses of benzenesufonyl chlorides (**2**) have recently been supported by theoretical calculations [7]. The calculations for water clusters in acetone indicate that reactions do not involve the formation of an intermediate and the S_N_3 reaction is favoured by electron withdrawing substituents and by clusters having a small number of water molecules (*i.e*., low *Y*).

**Figure 12:**
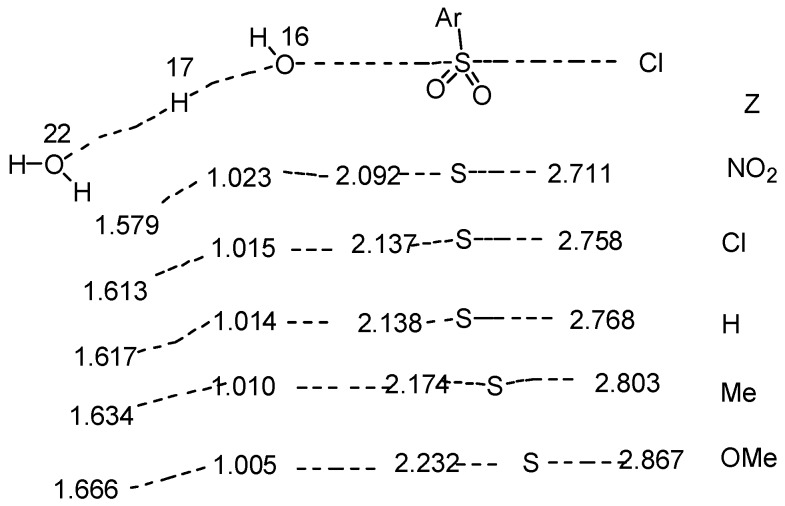
Calculated [7] changes in bond lengths (Angstroms) in transition states for hydrolyses of benzenesulfonyl chlorides (**2**) in a cluster of 17 water molecules in acetone.

The spectrum of mechanisms from nucleophilic attack by water (S_N_2), often assisted by general base catalysis by a second water molecule (S_N_3) can be illustrated (Figure 12) by the changes in bond lengths calculated [7] for a series of substrates (**2**, Z = OMe, Me, H, Cl and NO_2_).

The results, including numbering of atoms, are taken from Table 3 of Reference [7], and focus only on the substrate, the water molecule acting as nucleophile via O(16) and the water molecule acting as general base through O(22). Except for the approximately constant O(16)–H(17) distance, bond lengths extend significantly as the substituent changes from Z = NO_2_ to OMe; as expected for the proposed S_N_3 mechanism, the O(22)–H(17) distance in the second water molecule is considerably shorter than the value of 1.8 expected for a hydrogen bonded water molecule, and is shortest for Z = NO_2_.

### 2.6. Comments on Mechanistic Details

**Figure 13:**
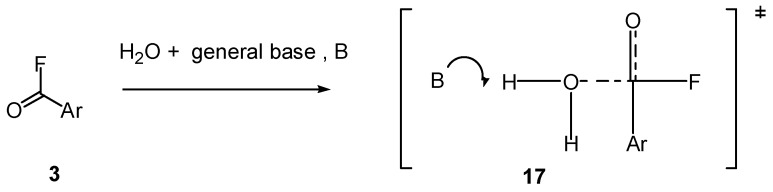
General base catalysis mechanism [9] for hydrolysis of benzoyl fluoride (**3**).

The mechanism of Song and Jencks [9] is illustrated in Figure 13 for benzoyl fluorides (**3**). The key features are the same as those shown in Figure 1b. General base catalysis by a base (B, e.g., water) assists nucleophilic attack by activating the attacking water nucleophile and by preventing the reverse reaction. The transition state could lead to a tetrahedral intermediate or there may be an “uncoupled concerted reaction” [9,88] in which the C–F bond is cleaved before the intermediate can be formed.

**Figure 14:**
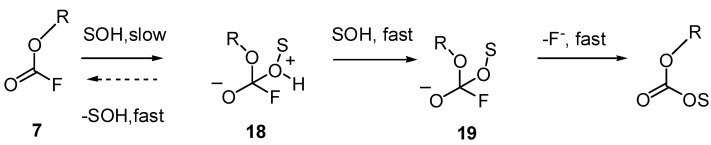
Stepwise addition-elimination mechanism [29,77,87,89] for solvolyses of haloformates, illustrated for nucleophilic attack by solvent (SOH) on fluoroformates (7).

In contrast, nucleophilic attack at the carbonyl group of chloro- and fluoroformates (**5**,**7**) is usually [29,77,87,89] described as shown in Figure 14. Two tetrahedral intermediates (**18**,**19**), not transition states are usually drawn. It is implied that there may be general base catalysis, but it not usually shown by these authors. However, general base catalysis is a key component of the proposed mechanism (Figure 1b and Figure 13), so the fast deprotonation step is avoided, and the first tetrahedral intermediate (**18**) is by-passed.

Also, it does not seem likely that the alkoxide intermediate (**19**) could be formed under typical reaction conditions of *ca.* 10^−2^ M substrate hydrolysed in unbuffered media, so the pH < 3; under these conditions, protonation of **19** would be expected, but the development of negative charge in the transition state is needed to explain the relatively high response to changes in *Y* for solvolyses of acid fluorides (Section 2.2.2 and Figure 3)*.* As well as the proposed [29] developing charge on oxygen, there may be a partial charge on fluorine in a transition state similar to **17**. F/Cl rate ratios of *ca.* 1 [29] provide evidence for the addition character of either a transition state or a tetrahedral intermediate [88]. 

Previously, it has been proposed that tetrahedral intermediates such as **19** may be by-passed during nucleophilic substitution reactions of acid chlorides [68] and chloroformates [90]. A single transition state has also been proposed for acyl group transfer between phenolate nucleophiles [91] and also for S_N_(Ar) reactions [92]. Therefore, although these reactions are often drawn to show stepwise mechanisms, there is substantial evidence for the concerted alternative.

The proposal that water as base assists water as nucleophile (Figure 1b) can be extended to hydroxide-catalysed hydrolyses of esters [93,94] and to S_N_(Ar) reaction [95]. For base-catalysed hydrolysis of methyl formate, the kinetic isotope effect on the oxygen nucleophile was explained [93] by water acting as nucleophile and hydroxide acting as general base. This mechanism was later supported by proton inventory measurements on the base-catalysed hydrolysis of ethyl acetate [94]. Alkoxide-catalysed hydrolysis of a chlorotriazine indirectly indicates that hydroxide-catalysed hydrolysis also occurs in S_N_(Ar) reactions [95].

## 3. Experimental Section

Kinetic data are from the references cited. Correlations were performed using Microsoft Excel.

## 4. Conclusions

Equation (3) provides a useful additional way to investigate mechanisms of hydrolyses, particularly for the S_N_2-S_N_3 spectrum (Table 1, Table 2 and Table 3) and for changes in reaction channel (e.g., Figure 6, Figure 7 , Figure 9, Figure 10 and Figure 11); it is more reliable than Equation (2) because solvent effects are included, and less complex than multi-parameter correlations (Section 2.4). KSIE data in water and methanol provide very useful additional information.

For sulfonyl chlorides, hydrolyses usually involve a second water molecule acting as a general base (S_N_2-S_N_3 channel), but there is no reliable evidence for an addition-elimination process involving an intermediate. For the most electron-rich substrates, Equation (3) plots (Figure 10 and Figure 11) support previous evidence for a gradual change in transition structure to the S_N_2-S_N_1 (cationic) reaction channel in the more polar mixtures; in less polar mixtures (lower *Y*), the S_N_2-S_N_3 channel becomes significant.

The same trends may apply to hydrolyses of other acid derivatives (e.g., haloformates and carboxylic acid halides), except that cationic reactions are more favourable for carboxylic acid halides. When the alternative “addition-elimination” process occurs (e.g., for fluoroformates), tetrahedral intermediates may be by-passed.
